# Supplementary material for: Content-rich biological network constructed by mining PubMed abstracts
Source: BMC Bioinformatics. 2004 Oct 8;5:147. doi: 10.1186/1471-2105-5-147 (PMC528731; doi:10.1186/1471-2105-5-147)
Supplement: Additional File 2 — The original results of the above study (non-essential files are deleted to keep the file size under the limit set by BMC bioinformatics). [file 1471-2105-5-147-S2.bz2 › chilibotAdditionalFile2/dip20/335ID8171323E3971/html/TNF_LTA.html]

 


 **TNF** and **LTA** 
  
Found 1446 abstracts in PubMed, retrieved 20.  
 

 What does Google say? 
 PDF only 
| .edu only 

---

**Interactive relationship** (e.g. stimulation, inhibition, etc)

**Neutral relationship**- In contrast, purified  **LTA**  triggered  **TNF**  release at 1 micro g ml.  Ref: 12819051 Infect Immun, 2003
- Analysis of the maturation process of dendritic cells deficient for  **TNF**  and lymphotoxin alpha  [ **LTA** ]  reveals an essential role for  **TNF** .  Ref: 12885938 J Leukoc Biol, 2003
- , Malvern, PA, USA, a chimaeric mAb, binds to soluble and membrane bound  **TNF**  alpha  [ **TNF** ] , but not to  **TNF**  beta  [ **LTA** ] .  Ref: 12718738 Expert Opin Biol Ther, 2003
- LT consists of soluble LT alpha  [ **LTA** ]  3, which binds to  **TNF**  R1 and  **TNF**  R2, and membrane LT alpha  [ **LTA** ]  1 beta 2, which binds to LT beta R.  Ref: 12882833 Int Immunol, 2003
- CONCLUSION The AA genotype at the lymphotoxin alpha  [ **LTA** ]  250 site is associated with higher serum tumor necrosis factor alpha  [ **TNF** ]  levels and a higher mortality in children with bacteremia.  Ref: 12780951 Pediatr Crit Care Med< MedlineTA>Pediatr Crit Care Med, 2003
- Samples homozygous at TNFA  [ **TNF** ]  308 and BAT1 intron 10 demonstrated identity between the African TNFA  [ **TNF** ]  308\*2 haplotype, the 8.1AH and the Asian diabetogenic 58.1AH HLA A33, B58, DR3 across a region spanning BAT1, ATP6G, IKBL,  **LTA** , TNFA  [ **TNF** ] , LTB, LST 1 and AIF 1.  Ref: 12859597 Tissue Antigens, 2003
- Serum tumor necrosis factor alpha  [ **TNF** ]  levels were 324 124 pg mL in bacteremic children with the AA genotype at the lymphotoxin alpha  [ **LTA** ]  250 site compared with 92 59 pg mL in bacteremic children with the AG genotype p < ;.  Ref: 12780951 Pediatr Crit Care Med< MedlineTA>Pediatr Crit Care Med, 2003
- Genetic polymorphisms located in the first intron of the lymphotoxin alpha  [ **LTA** ]  gene have been associated with increased secretion of tumor necrosis factor alpha  [ **TNF** ] .  Ref: 12780951 Pediatr Crit Care Med< MedlineTA>Pediatr Crit Care Med, 2003

**Non-interactive relationship** (e.g. studied together, co-existance, homology, etc.)

- Both Lymphotoxin alpha  [ **LTA** ]  and  **TNF**  Are Crucial for Control of Toxoplasma gondii in the Central Nervous System.  Ref: 12794148 J Immunol< MedlineTA>J Immunol, 2003
- METHODS In this study, we investigated an association between chronic periodontitis and two previously described bi allelic polymorphisms in the  **TNF**  locus a G to A transition at position 308 in the 5 promoter region of the  **TNF**  alpha  [ **TNF** ]  gene and an NcoI restriction fragment length polymorphism RFLP in the first intron position 252A G of the lymphotoxin alpha  [ **LTA** ]  LT alpha  [ **LTA** ]  gene.  Ref: 12828656 J Periodontal Res, 2003
- This activity of LT depends on membrane LT alpha  [ **LTA** ]  1 beta 2 and not soluble LT alpha  [ **LTA** ]  3, since DA virus persisted and induced inflammatory demyelination in LT beta R, but not  **TNF**  R1 or  **TNF**  R2, mice.  Ref: 12882833 Int Immunol, 2003
- Immunohistochemical and RT PCR analyses have shown elevated expression of pro inflammatory IL 6,  **TNF**  beta  [ **LTA** ] , ICAM 1 and pro fibrogenic TGF beta cytokines, and BMP 7 treatment significantly reduced their expression in the intestine.  Ref: 12811818 J Cell Physiol, 2003
- Polymorphisms in the 252 A G lymphotoxin alpha  [ **LTA** ]  and the 308 A G tumor necrosis factor alpha  [ **TNF** ]  genes and susceptibility to chronic periodontitis in a Czech population.  Ref: 12828656 J Periodontal Res, 2003
- However, the significant differences in the frequencies of the combined genotypes  **TNF**  alpha  [ **TNF** ]  and LT alpha  [ **LTA** ]  between the control and the patient groups were found using a simulation by applying the Monte Carlo method P <.  Ref: 12828656 J Periodontal Res, 2003
- **TNF**  alpha  [ **TNF** ]  and  **TNF**  beta  [ **LTA** ]  induced a 3 to 24 fold increase in IL 8 protein expression of BEC, and a 2 to 8 fold increased IL 8 expression in estrogen independent BCC cell lines and no significant IL 8 expression in estrogen dependent cell lines.  Ref: 12792762 Oncol Rep< MedlineTA>Oncol Rep, 2003
- CONCLUSION Our data suggest that combined genotypes composed of the  **TNF**  alpha  [ **TNF** ]  and LT alpha  [ **LTA** ]  gene polymorphisms may influence the susceptibility to chronic periodontitis.  Ref: 12828656 J Periodontal Res, 2003
- NcoI polymorphism exists in  **TNF**  gene promoter and influences  **TNF**  alpha  [ **TNF** ]  gene transcription.AIMTo determine the predictive value of plasma levels of  **TNF**  alpha  [ **TNF** ]  and its soluble receptors sTNFR,  **TNF**  alpha  [ **TNF** ]  308 and TNFB  [ **LTA** ]  polymorphisms on the occurrence of ASP and ASP associated early septic shock.METHODOLOGYGenotypes were determined in patients n = 208 and healthy controls n = 116 by means of restriction fragment length polymorphism analysis of polymerase chain reaction products.  Ref: 12717265 Pancreas, 2003
- DA persisted and induced inflammatory demyelination in LT alpha  [ **LTA** ]  but not  **TNF**  weanling mice of a normally resistant haplotype C57BL 6, suggesting that LT, but not  **TNF** , is critical for resistance to DA infection.  Ref: 12882833 Int Immunol, 2003
- Members of the tumour necrosis factor superfamily TNFSF  [ **LTA** ]  and  **TNF**  receptor superfamily TNFRSF have crucial roles in both innate and adaptive immunity.  Ref: 12860530 Trends Immunol, 2003
- Expression of cytokines  **TNF**  beta  [ **LTA** ]  and LT beta preceded, and the expression of TGF beta1 followed chemokine upregulation.  Ref: 12828562 Scand J Immunol, 2003
- Neutralizing antibodies against human RANTES, MIP 1alpha, MIP 1beta, alpha interferon IFN alpha, IFN beta, IFN gamma, interleukin 4 IL 4, IL 10, IL 13, IL 16, MCP 1, MCP 3, tumor necrosis factor alpha  [ **TNF** ]   **TNF**  alpha  [ **TNF** ] , or  **TNF**  beta  [ **LTA** ]  failed to reverse the HIV 1 suppressive activity.  Ref: 12885891 J VirolJ Virol, 2003
- Immunity to Toxoplasma gondii critically depends on TNFR type I mediated immune reactions, but the precise role of the individual ligands of TNFR1,  **TNF**  and lymphotoxin alpha  [ **LTA** ]  LTalpha, is still unknown.  Ref: 12794148 J Immunol< MedlineTA>J Immunol, 2003
- To analyze the involvement of tumor necrosis factor  [ **TNF** ]   **TNF**  and the related cytokine lymphotoxin LT alpha  [ **LTA** ]  in DC maturation, we studied the development of DCs from the BM of B6. **TNF** , B6.LTalpha, and B6. **TNF**  LTalpha mice and compared it to B6.WT mice.  Ref: 12885938 J Leukoc Biol, 2003
- We studied the biallelic interleukin 10 1082 promoter, the tumor necrosis factor alpha  [ **TNF** ]  308 promoter and the lymphotoxin alpha  [ **LTA** ]  polymorphisms with regard to the development of septic shock in pneumococcal infection.  Ref: 12746253 Am J Respir Crit Care Med< MedlineTA>Am J Respir Crit Care Med, 2003
- **TNF**  beta  [ **LTA** ]  and  **TNF**  alpha  [ **TNF** ]  308 polymorphisms were performed by analyzing NcoI digested DNA fragment obtained by polymerase chain reaction PCR.  Ref: 12888330 J Surg Res, 2003
